# Editorial: Brain Nutrient Sensing in the Control of Energy Balance: New Insights and Perspectives

**DOI:** 10.3389/fphys.2019.00051

**Published:** 2019-02-05

**Authors:** Céline Cruciani-Guglielmacci, Xavier Fioramonti

**Affiliations:** ^1^Unit of Functional and Adaptative Biology, Paris Diderot University, Paris, France; ^2^INRA UMR1286 Laboratoire NutriNeuro, Bordeaux, France

**Keywords:** nutrient sensing, energy balance, olfactory bulb, hypothalamus, aging

In the past decade the research on the homeostatic or non-homeostatic control of energy balance by the brain nutrient sensing has seen a profound evolution, and understanding the cellular and molecular mechanisms by which circulating nutrients act on brain have become a highly competitive field, with outcomes leading to several strategies to prevent or treat obesity and relative disorders. While studies on the role of hypothalamic networks have been the most numerous, more and more studies have recently described the crucial roles of other brain networks in the homeostatic and non-homeostatic control of energy metabolism. The present research topic proposes reviews and original articles covering this complex field of study of the importance of brain nutrient-sensing in the control of energy balance.

Glucose and lipids have been the most studied nutrient involved in brain nutrient-sensing. A Perspective Article with original data by Fioramonti et al. summarizes the recent advances in the cellular and molecular mechanisms of hypothalamic glucose detection. The authors pointed out the critical role of reactive oxygen species in glucose-sensing, and also discussed the newly discovered role of transient receptor potential canonical channels (TRPC) in glucose detection. In addition, they pointed out the possible implication of metabolism-independent glucose-sensing mechanisms through either the sodium-glucose cotransporter (SGLT) or sweet taste receptors. This article, thanks to original data, helped understanding the controversy around the direct glucose-sensing properties of the melanocortin neurons of the arcuate nucleus of the hypothalamus. Zhou et al. presented another Perspective Article, on the role of hypothalamic glucose-inhibited (GI) neurons in the detection and regulation of hypoglycaemia, which is a profound threat to the brain since glucose is its primary fuel. Their discussed the features of GI neurons in the ventromedial hypothalamus (VMH), which expresses neuronal nitric oxide synthase (nNOS), and in the perifornical hypothalamus (PFH) which expresses orexin. They then highlighted new interesting approaches to prevent hypoglycaemia: to enhance antioxidant systems (such as glutathione or thioredoxin), to explore how orexin-GI neurons play a role in stimulating food intake (which leads to a corrected glycemia), and the relationships between hypothalamic sensors and hindbrain neurocircuitry… Finally, an original article from Yuan et al. on extra-hypothalamic glucose-sensing networks demonstrated the role of Nesfatin-1 neurons in the lateral parabrachial nucleus (LPBN) in food intake and energy expenditure controls. This effect is mediated through the excitation of most of the glucose-inhibited neurons in the LPBN, which leads to enhanced UCP1 expression in brown adipose tissue via the melanocortin system.

Two Review Articles treated different impact of lipid-sensing on either energy homeostasis or mood and cognitive functions. Larrieu and Layé addressed the question of a link between dietary omega-3 polyunsaturated fatty acids and neuropsychiatric diseases such as anxiety and depression. They discussed potential mechanisms involved in the neuroprotective and corrective activity of ω3 PUFAs in the brain, in particular the sensing activity of free fatty acid receptors, the activity of the PUFAs-derived endocannabinoid system and the hypothalamic-pituitary adrenal axis. Among lipids, several studies reviewed by Cruciani-Guglielmacci et al. suggested that ceramides could be involved in the regulation of energy balance in both hypothalamic and extra-hypothalamic areas. Interestingly, under lipotoxic conditions, these ceramides were shown to play a role in the dysregulation of central control of glucose homeostasis. Thus, pushing ceramide metabolism toward the synthesis of less harmful lipids, such as Sphingosine 1-phosphate, with the use of sphingosine kinase 1 activators could represent a new therapeutic approach to counteract lipotoxicity.

The impact of nutrients and energy status of olfactory functions and, reciprocally, the impact of this system on food intake is a fairly new field of investigation arousing the scientific committee. In the present topic, two original research articles and one review article highlighted the role of the olfactory bulb (OB) in the control of food intake and whole body metabolism. In particular Kovach et al. explored the role of the potassium channel Kv1.3, whose deletion in the OB lead to “super-smeller mice,” on mitochondrial structure and glucose utilization. These changes are initiated at the OB level, but they could drive whole system changes in metabolism. Another team, Chelminski et al., questioned the role of leptin receptors in the OB, suggesting a role for leptin in odor-evoked activities. They showed that cellular dynamics in the OB of leptin-deficient mice (ob/ob) are deeply modified in the context of olfactory learning. The review by Julliard et al. emphasized the link between nutrient sensing and the olfactory system. They first recalled that orexigenic peptides such as ghrelin and orexin increase olfactory sensitivity, which in turn, is decreased by anorexigenic hormones such as insulin and leptin. Then, they summarized recent findings on nutrient sensing in the OB. This new line of investigation at the crossroad between olfaction and food behavior could contribute to better determining the etiology of metabolic disorders.

Finally, a review article by de Lucia et al. highlighted the emerging molecular pathways that govern the dietary regulation of neural stem cells (NSCs) during aging. They discussed the importance of the Sirtuin, mTOR and Insulin/Insulin like growth factor-1 pathways as well as the significant role played by epigenetics in the dietary regulation of NSCs. Taking all the data together, nutrition may be a promising mode of intervention to regulate NSCs and prevent the cognitive decline associated with aging.

Overall, the present topic, through original or perspective articles and reviews, gives a clear view on the current question addressed by the scientific community working on brain glucose-sensing, and opens to new questions which still need to be addressed to fully understand the impact of nutrient-sensing, and more generally nutrition, on brain functions.

## Author Contributions

All authors listed have made a substantial, direct and intellectual contribution to the work, and approved it for publication.

### Conflict of Interest Statement

The authors declare that the research was conducted in the absence of any commercial or financial relationships that could be construed as a potential conflict of interest.

